# Cerebrovascular regulation dynamics and Alzheimer's neuroimaging phenotypes

**DOI:** 10.1002/alz.71146

**Published:** 2026-02-13

**Authors:** Amaryllis A. Tsiknia, Jamie A. Terner, Zoe E. Tsokolas, Dae C. Shin, Elizabeth B. Joe, Peter S. Conti, Rebecca J. Lepping, Brendan J. Kelley, Rong Zhang, Sandra A. Billinger, Helena C. Chui, Vasilis Z. Marmarelis, Meredith N. Braskie

**Affiliations:** ^1^ Mark and Mary Stevens Neuroimaging and Informatics Institute University of Southern California Los Angeles California USA; ^2^ Biomedical Engineering Department University of Southern California Los Angeles California USA; ^3^ Department of Neurology Keck School of Medicine University of Southern California Los Angeles California USA; ^4^ Alzheimer's Disease Research Center Keck School of Medicine University of Southern California Los Angeles California USA; ^5^ Molecular Imaging Center Department of Radiology Keck School of Medicine University of Southern California Los Angeles California USA; ^6^ Department of Neurology University of Kansas Medical Center Kansas City Kansas USA; ^7^ Department of Neurology UT Southwestern Medical Center Dallas Texas USA; ^8^ Institute for Exercise and Environmental Medicine Texas Health Presbyterian Hospital Dallas Texas USA

**Keywords:** Alzheimer's disease, amyloid, autoregulation, biomarkers, cerebral blood flow, cerebral hemodynamics, cerebrovascular function, cerebrovascular reactivity, MRI, near‐infrared spectroscopy, neuroimaging, PET, transcranial doppler ultrasound

## Abstract

**INTRODUCTION:**

Cerebrovascular dysfunction may contribute to Alzheimer's disease (AD) pathogenesis. We examined how novel cerebral hemodynamic markers relate to neuroimaging phenotypes associated with AD dementia in cognitively impaired and unimpaired older adults.

**METHODS:**

Statistical hemodynamic indices were computed for each participant from stochastic dynamic models relating resting‐state spontaneous arterial blood pressure and end‐tidal CO_2_ fluctuations to transcranial doppler‐derived blood velocity and near infrared spectroscopy‐derived cortical tissue oxygenation. Linear regressions related these hemodynamic indices to hippocampal volume, WMH volume, cortical thickness in an AD‐signature region, and brain amyloid burden measured by PET.

**RESULTS:**

Higher hemodynamic indices, indicating proximity to normal cerebrovascular function correlated with neuroimaging phenotypes typically associated with better cognitive status: greater hippocampal volume and lower amyloid burden.

**DISCUSSION:**

Our findings provide further support for the role of cerebrovascular dysfunction in AD pathogenesis and for the potential clinical utility of model‐based indices of cerebral hemodynamics.

## Highlights

- Model‐based hemodynamic marker estimation involves no effort from the participant.
- All hemodynamic indices differ between cognitively impaired and unimpaired adults.
- Novel hemodynamic markers correlate with established ADRD‐related brain phenotypes.
- Near‐infrared spectroscopy‐derived indices correlate with hippocampal volume.
- Dynamic cerebral autoregulation correlates with amyloid burden.

## 1 INTRODUCTION

Alzheimer's disease and related dementias (ADRDs) are progressive neurological conditions with underlying pathological processes that begin decades before clinical symptoms emerge^1^, ^2^. Cerebrovascular dysfunction may be one of the earliest such changes^3^. Therefore, detecting early cerebrovascular abnormalities may help identify individuals at risk and facilitate timely intervention. Measures of cerebral hemodynamics may be potential candidate biomarkers of early cerebrovascular dysfunction associated with mild cognitive impairment (MCI) and dementia^4^, ^5^. The term cerebral hemodynamics encompasses the dynamic regulatory mechanisms that ensure the brain parenchyma receives the oxygen and nutrients necessary to meet fluctuating metabolic demands under varying physiological conditions^6^, ^7^. Specifically, cerebral autoregulation describes the capacity of the vasculature to maintain constant brain perfusion in spite of perfusion pressure changes^8^. Cerebrovascular reactivity to CO_2_ describes the ability of the vasculature to dilate or constrict in order to increase (or decrease) perfusion in response to a rise (or drop) in the partial pressure of arterial CO_2_, in order to match the metabolic needs of the brain tissue^9^.

While cerebral autoregulation is preserved in MCI and dementia^10^, ^11^, ^12^, ^13^ cerebrovascular reactivity to CO_2_ is reduced^14^, ^15^, ^16^, ^17^, ^18^ correlating with cognitive decline^19^, ^20^. The vascular hypothesis of AD posits that vascular dysfunction contributes to disease pathogenesis^21^, ^22^. Indeed, vascular risk factors, such as hypertension, are linked to greater ADRD risk,^23^ and hypertension treatment is associated with reduced dementia incidence^24^. Many mechanisms linking vascular dysfunction to ADRD converge on cerebral hypoperfusion, which can lead to hypoxia, neuroinflammation, blood‐brain barrier damage, and neurodegeneration^25^. Hypoperfusion and hypoxia may also directly accelerate the accumulation of amyloid‐β in the brain^26^, ^27^. However, human studies examining how cerebral hemodynamics relate to neuroimaging markers associated with ADRD are limited.

Our study addresses this knowledge gap by examining how model‐based indices of cerebral hemodynamics relate to established ADRD neuroimaging endophenotypes in older adults across the cognitive continuum. We employ a novel model‐based approach^28^ that uses transcranial doppler ultrasound to measure cerebral blood velocity and near‐infrared spectroscopy to measure cortical tissue oxygenation at rest in response to spontaneous fluctuations in arterial blood pressure and CO_2_ tension. Unlike conventional techniques involving CO_2_ inhalation, breath‐holding and blood pressure manipulation,^29^, which rely heavily on participant compliance, this approach involves no effort on behalf of the participant.

This technique uses principal dynamic mode decomposition to characterize the dynamic relationships between two physiological inputs–namely, arterial blood pressure or end‐tidal CO_2_ and two physiological outputs of interest–namely cerebral blood velocity and cortical tissue oxygenation. Using this approach we derive five distinct hemodynamic indices: (1) dynamic vasomotor reactivity (DVR), which captures how cerebral blood velocity changes in response to spontaneous fluctuations in end‐tidal CO_2_, (2) dynamic cerebral autoregulation (DCA) which reflects how cerebral blood velocity changes in response to blood pressure fluctuations, (3) cortical oxygenation CO_2_ reactivity (COCR), which captures cortical tissue oxygenation changes in response to spontaneous end‐tidal CO_2_ fluctuations, (4) cortical oxygenation pressure reactivity (COPR), which captures cortical tissue oxygenation changes in response to arterial blood pressure fluctuations, and finally (5) a composite cortical oxygenation reactivity (composite‐COR) index integrating COCR and COPR. Importantly, DVR and COCR reflect vascular reactivity to CO_2_, at the level of cerebral blood velocity and tissue oxygenation, respectively. Similarly, DCA and COPR reflect cerebral autoregulation to blood pressure at the level of cerebral blood velocity and tissue oxygenation, respectively. These indices have distinguished individuals with MCI or AD dementia from cognitively unimpaired controls^30^, ^31^ highlighting their clinical relevance for detecting cerebrovascular dysfunction in neurodegenerative conditions.

**RESEARCH IN CONTEXT**

1. **Systematic review**: We searched PubMed for human studies examining cerebral hemodynamics and their association with ADRD‐related brain changes. Relevant studies were limited and involved conventional experimental techniques for probing cerebral hemodynamics that require participants to perform patterned breath‐holding or manipulation of blood pressure.

2. **Interpretation**: Our novel model‐based approach leverages spontaneous fluctuations in arterial blood pressure and CO2 tension without interventions. Impairments in cerebral hemodynamic properties probed using this method are associated with established ADRD‐related neuroimaging phenotypes – smaller hippocampal volume and greater amyloid burden – providing further evidence for an important role of cerebrovascular dysfunction in the development of ADRD.

3. **Future directions**: Future studies should examine whether novel, model‐based cerebral hemodynamic markers can accurately predict future cognitive impairment, and longitudinal changes in brain structure and ADRD neuropathology.

Here, we examine how these novel markers of cerebral hemodynamics relate to established ADRD‐related neuroimaging measures, including hippocampal volume, cortical thickness in a composite of temporal AD‐signature brain regions,^32^ white matter hyperintensity (WMH) volume, and cerebral amyloid burden. We hypothesize that higher values for hemodynamic indices, indicative of cerebrovascular function that is more typical of cognitively unimpaired individuals, will be associated with more favorable neuroimaging profiles across modalities.

## 2 METHODS

### 2.1 Participants

Participants were recruited from existing Alzheimer's Disease Research Center clinical core cohorts at the University of Southern California (USC) and at the University of Kansas Medical Center (KUMC), affiliated memory clinics at USC and at the University of Texas Southwestern (UTSW), and ongoing site‐specific community outreach efforts across sites.

At the time of the analysis, 206 study participants had undergone near infrared spectroscopy (for measurement of COCR, COPR, and composite‐COR) and 167 had undergone transcranial doppler ultrasound (for DVR and DCA measurement). There were fewer people with available DVR/DCA data than COCR/COPR/composite‐COR data due to the inability in some participants to detect a signal using the temporal window for middle cerebral artery blood velocity.

Of the 206 older adults with COCR, COPR, and composite‐COR data, 161 participated in baseline MRI and 136 underwent a baseline 18F‐florbetaben PET scan. Of the 167 participants with DVR and DCA data, 135 completed an MRI scan and 123 underwent a florbetaben PET scan. We excluded participants on a per‐imaging modality basis due to either 1) an incidental finding that would bias the processing/analyses, 2) missing scan sequences if the participant was unable to tolerate the whole protocol, or 3) unusable data because of either a scan artifact or a failure of an analysis step. See Figures S1 and S2 for further details on sample size by each neuroimaging modality and see Table S1 for details regarding the overlap between samples.

### 2.2 Data collection

MRI scans, PET scans, and vascular measures were collected at separate study visits. The time elapsed between MRI scans and vascular visits ranged from 0 to 10 months (mean = 0.79 months), and the time elapsed between PET scans and vascular visits ranged from 0 to 14 months (mean = 1.53 months). Visits took place across three study sites: UTSW, KUMC, and USC. Each participant attended all study visits at the same site.

### 2.3 MRI acquisition and processing

Participants from USC and UTSW were scanned on a 3T Siemens Magnetom Prisma, and participants from KUMC were scanned on a 3T Siemens Magnetom Skyra. Cortical thickness and hippocampal volume analyses were performed on T1‐weighted magnetization‐prepared rapid acquisition gradient echo (MPRAGE) images (TR = 2300 ms; TE = 2.98 ms; 1.00 mm slices; 1.0 × 1.0 × 1.0 mm voxel size for all scanners, and inversion time = 900 ms). WMH analyses were performed using T2‐weighted fluid attenuated inversion recovery (FLAIR) images (USC and UTSW: TR = 4800 ms; TE = 441 ms; 1.20 mm slices; inversion time = 1550 ms; 1.0 × 1.0 × 1.2 mm voxel size; KUMC: TR = 5000 ms; TE = 388 ms; 1.00 mm slices; inversion time = 1800 ms; 1.0 × 1.0 × 1.0 mm voxel size).

We reoriented all T1‐weighted MPRAGE images to the standard MNI152 template orientation using the fslreorient2std and robustfov functions of the FMRIB software library (FSL). We then used the advanced normalization tool (ANTs) to perform N4 bias correction and used FreeSurfer (version 5.3.0) to automatically segment the N4‐corrected images into gray and white matter and to parcellate 34 cortical regions on the right and left hemisphere, for a total of 68 cortical regions. The bilateral entorhinal, fusiform, inferior and middle temporal gyri segmentations were used to create an AD‐signature meta‐ROI for cortical thickness analysis^32^. We quality checked the segmentation of each cortical region using a modified version of the ENIGMA internal quality check pass‐fail protocol (see Supporting Information for more details). HippoDeep software deepseg1 was used to segment the right and left hippocampus and to estimate intracranial volume. We quality checked these segmentations by following the recommendations of the European Alzheimer's Disease Consortium and the AD Neuroimaging Initiative harmonized hippocampal protocol (see Figures S3, S4, and S5 for a depiction of the hippocampal mask, and examples of oversegmentation and undersegmentation, respectively)^33^. A bilateral hippocampal volume measure was computed as the average of left and right hippocampal volume for statistical analysis.

WMH were segmented by the lesion growth algorithm as implemented in the Lesion Segmentation Tool (LST) toolbox (www.statisticalmodelling.de/lst.html) for SPM^34^. The optimal initial threshold was determined by visual inspection and ultimately set at a kappa value of 0.3. This algorithm relied on input from each participant's T1 and FLAIR images. We visually quality checked lesion segmentations using an in‐house pass‐fail protocol based on how accurately the segmentations captured the extensiveness of the lesions.

### 2.4 PET acquisition and processing

Amyloid PET imaging was performed across all sites using a florbetaben (Neuraceq) radiotracer injection at 300 MBq (8.1 mCi) ± 10%. At 90 min post‐injection, 20 min dynamic scans consisting of four 5 min frames were acquired on a GE Medical Systems Discovery MI DR (KUMC), GE Medical Systems Discovery MI Five Ring (UTSW), or Siemens Biograph TruePoint (USC). Images were reconstructed and reviewed for excessive motion or artifact immediately following the PET scan. KUMC images were reconstructed using a 3D iterative VHPD method, consisting of 6 iterations and 16 subsets (Grid = 192 × 192; FOV = 256 mm; 2.79 mm slices; no smoothing filter). USC scans were reconstructed using an iterative OSEM‐3D method, consisting of 4 iterations and 21 subsets (Grid = 336 × 336 × 109; zoom = 2.0; 2.027 mm slices; no smoothing filter). UTSW scans were reconstructed with an iterative VPHD method, consisting of 3 iterations and 34 subsets (Grid = 128 × 128; 2.027 mm slices; no smoothing filter). The Alzheimer's Disease Neuroimaging Initiative 3 (ADNI3) protocol was used for the acquisition and analysis of all amyloid PET scans,^35^.

The protocol for analysis of amyloid PET scans included motion correction between frames using the SPM12 toolbox of MATLAB, resampling of the mean data across the co‐registered images to create one volume with isometric (1.5 mm) voxels, applying a full width half maximum (FWHM) smoothing kernel (KUMC: in‐plane = 5.5 mm and axial = 5.0; USC: in‐plane = 5.5 mm and axial = 5.5 mm; UTSW: in‐plane = 6.0 mm and axial = 5.5 mm) to smooth to the 8 mm full‐width‐half‐maximum per ADNI3 protocols for florbetaben scans, and finally performing a series of linear registrations between the T1‐weighted MPRAGE and associated FreeSurfer segmentations to the participant‐specific amyloid PET scans. Registration of each participant's PET scan to their T1‐weighted MRI scan was performed using FSL FLIRT with six degrees of freedom and a mutual information cost function. The inverse of these transformation matrices was then applied to the ROIs and reference regions to map them back into PET space, where all analyses were performed^36^. To derive a measure of global amyloid burden, we first calculated the SUVR in four FreeSurfer‐derived cortical regions (frontal, anterior/posterior cingulate, lateral parietal, and lateral temporal lobes) by dividing the mean PET signal in that region by the mean PET signal in a FreeSurfer‐defined cerebellum reference region and then computed the straight average of those four SUVRs.

### 2.5 Acquisition and analysis of cerebral hemodynamic measures

While participants were resting in the supine position, for eight minutes we continuously measured arterial blood pressure using finger photo‐plethysmography (Finapres), end‐tidal CO_2_ via a nasal cannula using capnography (Criticare Systems), and heart rate using electrocardiography. A 2 MHz transcranial doppler probe (Multiflow, DWL) was placed over the temporal window to measure middle cerebral artery velocity. Near‐infrared spectroscopy detection probes (Hamamatsu) were placed over the lateral prefrontal cortex to measure tissue oxygenation (oxyhemoglobin/total hemoglobin × 100). Study personnel responsible for data collection followed a strict standard operating procedure that was established during the study start‐up phase. This protocol has previously demonstrated good test‐retest reproducibility in 70 cognitively normal older adults, with intra‐trial coefficients of variation below 10% across physiological signals (7.7% for middle‐cerebral artery velocity, 6.7% for arterial blood pressure, and 8.6% for end‐tidal CO_2_)^37^. Additionally, standardized procedures were implemented for the concurrent acquisition of transcranial doppler ultrasound and near‐infrared spectroscopy measurements across all sites. All data collection staff underwent rigorous training involving 3‐4 supervised training sessions with participant actors and achieved an intra‐class correlation coefficient of 0.9 or higher compared to an established expert before moving forward with independent data collection.

Preprocessing and analysis methods have been described previously^20^ and are detailed in the Appendix. Briefly, physiological measurements were visually inspected and signal outliers (± 3 SD around the time‐averaged value) were removed. Beat‐to‐beat blood pressure, cerebral blood velocity and tissue oxygenation data were synchronized with breath‐to‐breath end‐tidal CO_2_ data using cubic spline interpolation, and frequencies below 0.005 Hz were removed. A two‐input linear dynamic model was used to express cerebral blood velocity or cortical tissue oxygenation in terms of the convolutional relation between blood pressure and the CO_2_ time‐series data. Their respective kernels were then estimated using the Laguerre expansion technique. We then performed singular value decomposition on each kernel to derive a set of orthonormal principal dynamic modes, ranked by their singular values. Subsequently, the expansion coefficients of each kernel on the respective orthonormal principal dynamic mode basis were computed via inner products and termed “principal dynamic mode gains”. These principal dynamic mode gains were used to compute log‐likelihood ratios, indicating how closely a participant's dynamics matched those of the cognitively impaired vs. unimpaired groups. Positive log‐likelihood ratios indicate proximity of the principal dynamic mode gain to the cognitively unimpaired group and negative log‐likelihood ratios indicate proximity to the cognitively impaired group. Each hemodynamic marker was computed as the weighted sum of the log‐likelihood ratios of all principal dynamic gains for the respective input‐output pathway, with the weights determined through regression and held constant across participants. Log‐likelihood ratios were determined separately for *APOE* ε4 carriers and non‐carriers, since the presence of the *APOE* ε4 allele significantly affects cerebral hemodynamics.

We also computed physiological “raw” versions of those hemodynamic indices (rather than log likelihood values). Physiological hemodynamic indices were computed as the integral of the kernel corresponding to the step‐response of each output to the respective input (i.e. the step‐response of cerebral blood velocity to end‐tidal CO_2_ fluctuations for DVR, and to arterial blood pressure fluctuations for DCA, and the step‐response of cortical tissue oxygenation to arterial blood pressure fluctuations for COPR, and to end‐tidal CO_2_ fluctuations for COCR).

### 2.6 Clinical evaluations

All participants underwent a clinical evaluation using version 3 of the National Alzheimer's Coordinating Center's Uniform data set,^38^ assessments, including the clinical dementia rating and neuropsychological testing, and were categorized by clinicians at each study site as being cognitively normal or having MCI or mild dementia, according to standard protocols.

### 2.7 Statistical analysis

Statistical analyses were performed in RStudio (version 2022.07.2). First, we used a one‐way ANOVA and a Tukey's Honestly Significant Difference test to examine differences in hemodynamic markers across the three clinical groups (CN, MCI, and dementia).

We then performed separate multiple linear regressions relating each of the hemodynamic markers (DVR, DCA, COCR, COPR, and composite‐COR) to each of the neuroimaging measures of interest (WMH volume, meta‐ROI cortical thickness, global amyloid SUVR, and hippocampal volume). All linear regression models included covariates for age, self‐reported sex, study site, *APOE* ε4 status, and total Montreal Cognitive Assessment (MoCA) score. Because cognitive diagnosis was highly collinear with the hemodynamic indices and the neuroimaging measures, we covaried for MoCA (instead of cognitive diagnosis) in all primary models. Regression models of WMH and hippocampal volume additionally covaried for intracranial volume. In the case of a significant association between a hemodynamic index and a neuroimaging measure, we stratified our sample by cognitive status and tested for the association in cognitively impaired and unimpaired participants separately. To formally test whether cognitive performance moderated these associations, we also examined interaction terms between continuous total MoCA scores and our hemodynamic measures on the neuroimaging measures in the full sample. Additionally, using a linear regression model with covariates for age and sex, we examined the relationship between each of the hemodynamic measures and total MoCA scores.

To further probe significant associations between hemodynamic indices and neuroimaging measures, we additionally performed a sensitivity analysis using linear regressions relating the physiological versions of those hemodynamic indices (rather than log likelihood values) to neuroimaging outcomes using all the same covariates.

All continuous variables were normalized to obtain z‐scores, and all reported linear regression model parameter estimates are standardized. We used the Bonferroni method to correct for comparisons across the four independent hemodynamic measures (DVR, DCA, COCR, and COPR). Consequently, p‐values < 0.013 were regarded as statistically significant.

Eight participants had measurements taken from the left rather than the right middle cerebral artery. To determine whether the hemisphere of transcranial doppler ultrasound acquisition influenced DVR or DCA values, we conducted a linear regression analysis with DVR or DCA values as the output measure and recording hemisphere, age, and sex as the predictors. Because the side of transcranial doppler acquisition did not significantly influence DVR values (β = 0.19, 95% CI: [‐0.159, 0.548], p > 0.10) or DCA values (β = 0.01, 95% CI: [‐0.297, 0.321], p > 0.10), we did not include hemisphere as a covariate in subsequent DVR and DCA analyses.

## 3 RESULTS

### 3.1 Participant sample characteristics

In the overall sample of 133 participants with a DVR and DCA measurement and usable data for at least one neuroimaging modality, participants were on average 73 years old, 63 were female, 61 were CN, 45 had MCI, and 27 had dementia. Complete demographic and baseline clinical information for the DVR and DCA sample can be found in Table 1.

**TABLE 1:** Demographic and clinical characteristics of 133 participants with DVR and DCA data.

Characteristic	Overall, *N* = 133^1^
**Age**	73 (8)
**Sex**	
Female	63 (47%)
Male	70 (53%)
**Education**	17 (3)
(Missing)	13
**Hypertension**	
Not present	65 (54%)
Present	55 (46%)
(Missing)	13
**Cognitive Diagnosis**	
CN	61 (46%)
MCI	45 (34%)
Dementia	27 (20%)
**Site**	
KUMC	60 (45%)
USC	51 (38%)
UTSW	22 (17%)
**DVR**	−0.05 (0.49)
**DCA**	−0.03 (0.43)
**MoCA total score**	22.70 (5.75)
(Missing)	16
**Ethnicity**	
Hispanic	7 (5.3%)
Non‐Hispanic	124 (95%)
(Missing)	2
**Race**	
Asian	2 (1.5%)
Black or African American	7 (5.4%)
Native Hawaiian or Other Pacific Islander	1 (0.8%)
Other	3 (2.3%)
Unknown	2 (1.5%)
White	115 (88%)
(Missing)	3
***APOE* ε4 status**	
Carrier	55 (41.3%)
Non‐carrier	78 (58.6%)

^1^ Mean (SD); *n* (%).

In the overall COCR/COPR/composite‐COR sample of 159 older adults with usable data for at least one neuroimaging modality, participants were on average 73 years old, 81 were female, 71 were CN, 52 had MCI and 36 had dementia. Details on demographic and baseline clinical characteristics for the COCR, COPR, and composite‐COR sample are shown in Table 2.

**TABLE 2:** Demographic and clinical characteristics of 159 participants with COCR, COPR, and composite‐COR data.

Characteristic	Overall, *N* = 159^1^
**Age**	73 (8)
**Sex**	
Female	81 (51%)
Male	78 (49%)
**Education**	17 (3)
(Missing)	17
**Hypertension**	
Present	66 (46%)
Not present	76 (54%)
(Missing)	17
**Cognitive Diagnosis**	
CN	71 (45%)
MCI	52 (33%)
Dementia	36 (23%)
**Site**	
KUMC	64 (40%)
USC	70 (44%)
UTSW	25 (16%)
**COCR**	−0.05 (0.53)
**COPR**	−0.10 (0.59)
**Composite‐COR**	−0.13 (0.72)
**MoCA total score**	22.46 (6.06)
(Missing)	20
**Ethnicity**	
Hispanic	15 (9.6%)
Non‐Hispanic	142 (90%)
(Missing)	2
**Race**	
White	131 (84%)
Black or African American	8 (5.1%)
Asian	6 (3.8%)
Native Hawaiian or Other Pacific Islander	1 (0.6%)
Other	7 (4.5%)
Unknown	3 (1.9%)
(Missing)	3
***APOE* ε4 status**	
Carrier	65 (40.9%)
Non‐carrier	94 (59.1%)

^1^ Mean (SD); *n* (%).

### 3.2 Differences in hemodynamic indices between cognitive groups

**FIGURE 1:**
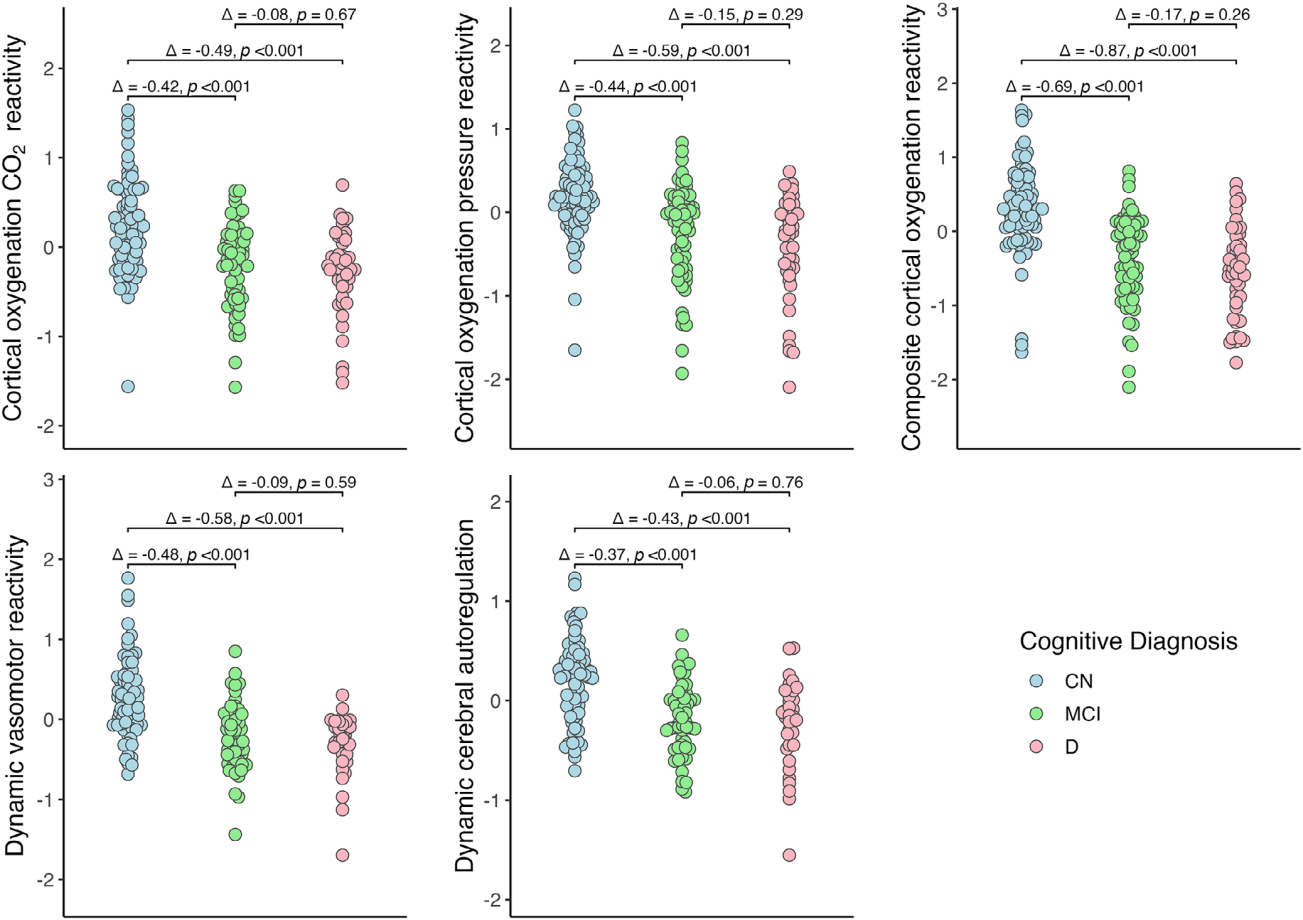
Title. differences in hemodynamic indices across clinical groups.

CN participants had the highest values on all hemodynamic measures (DVR: mean = 0.26, SD = 0.48; DCA: mean = 0.21, SD = 0.41; COCR: mean = 0.21, SD = 0.49; COPR: mean = 0.20, SD = 0.43; composite‐COR: mean = 0.32, SD = 0.57), participants with MCI had intermediate values (DVR: mean = ‐0.22, SD = 0.41; DCA: mean = ‐0.17, SD = 0.36; COCR: mean = ‐0.21, SD = 0.45; COPR: mean = ‐0.24, SD = 0.56; composite‐COR: mean = ‐0.37, SD = 0.59), and participants with dementia had the lowest values (DVR: mean = ‐0.32, SD = 0.39; DCA: mean = ‐0.23; SD = 0.44; COCR: mean = ‐0.28, SD = 0.45; COPR: mean = ‐0.39, SD = 0.61; composite‐COR: mean = ‐0.55, SD = 0.60). Hemodynamic indices in CN participants were significantly greater than those in both participants with MCI and participants with dementia, but MCI and dementia groups did not differ significantly. Cognitive group differences in hemodynamic measures are shown in Figure 1.

### 3.3 Associations between hemodynamic indices (DVR, DCA, COCR, COPR, and composite‐COR) and neuroimaging measures (hippocampal volume, global amyloid burden, WMH volume, and cortical thickness in a temporal AD‐signature region)

All associations between hemodynamic indices and neuroimaging markers are summarized in Table 3.

**TABLE 3:** Results of linear regression models relating each hemodynamic index to each neuroimaging outcome, while covarying for age, sex, site, total MoCA score, and *APOE* ε4 as well as intracranial volume for analyses that use WMH or hippocampal volume as outcomes.

	WMH volume	Meta‐ROI cortical thickness	Hippocampal volume	Global amyloid SUVR
DVR	β = ‐0.20, 95% CI: [‐0.38, ‐0.03], *p* **= 0.02**, *N* = 116	β = ‐0.008, 95% CI: [‐0.19, 0.18], *p* > 0.10, *N* = 66	β = 0.08, 95% CI: [‐0.08, 0.24], *p* > 0.10, *N* = 109	β = ‐0.12, 95% CI: [‐0.30, 0.06], *p* > 0.10, *N* = 99
DCA	β = 0.02, 95% CI: [‐0.16, 0.20], *p* > 0.10, *N* = 116	β = ‐0.05, 95% CI: [‐0.24, 0.15], *p* > 0.10, *N* = 66	β = 0.17, 95% CI: [0.02, 0.32], ***p* = 0.03**, *N* = 109	β = ‐0.23, 95% CI: [‐0.41, ‐0.05], *p* = 1.12e‐02^*^, *N* = 99
COCR	β = ‐0.09, 95% CI: [‐0.26, 0.08], *p* > 0.10, *N* = 136	β = 0.15, 95% CI: [‐0.02, 0.32], *p* = 0.08, *N* = 76	β = 0.18, 95% CI: [0.05, 0.32], *p* = 7.74e‐03^*^, *N* = 130	β = ‐0.19, 95% CI: [‐0.35, ‐0.02], ***p* = 0.03**, *N* = 113
COPR	β = ‐0.05, 95% CI: [‐0.23, 0.13], *p* > 0.10, *N* = 136	β = 0.20, 95% CI: [0.03, 0.37], ***p* = 0.02**, *N* = 76	β = 0.23, 95% CI: [0.08, 0.38], *p* = 3.73e‐03^*^, *N* = 130	β = ‐0.24, 95% CI: [‐0.42, ‐0.07], *p* = 5.65e‐03^*^, *N* = 113
Composite‐COR	β = ‐0.06, 95% CI: [‐0.24, 0.13], *p* > 0.10, *N* = 136	β = 0.18, 95% CI: [0.01, 0.36], ***p* = 0.04**, *N* = 76	β = 0.26, 95% CI: [0.11, 0.41], *p* = 6.21e‐04^*^, *N* = 130	β = ‐0.25, 95% CI: [‐0.43, ‐0.08], *p* = 5.15e‐03^*^, *N* = 113

*p* < 0.05 bold, *p* < 0.013 (significant after Bonferroni correction) bold with asterisk.

Higher DVR was nominally associated with lower WMH volume, but this was not significant after multiple comparisons correction (β = ‐0.20, 95% CI: [‐0.38, ‐0.03], *p* = 0.02). There was no association between DVR and meta‐ROI cortical thickness, hippocampal volume or global amyloid SUVR (Table 3).

Higher DCA correlated significantly with lower amyloid burden (β = ‐0.23, 95% CI: [‐0.41, ‐0.05], p = 1.12e‐02) and at a trend‐level with greater hippocampal volume (β = 0.17, 95% CI: [0.02, 0.32], *p* = 0.03). There was no association between DCA and WMH volume or meta‐ROI cortical thickness (Table 3).

Higher COCR was significantly associated with greater hippocampal volume (β = 0.18, 95% CI: [0.05, 0.32], *p* = 7.74e‐03), and at a trend‐level with lower global amyloid SUVR (β = ‐0.19, 95% CI: [‐0.35, ‐0.02], *p* = 0.03), but not with WMH volume, or meta‐ROI cortical thickness (Table 3).

Higher COPR correlated significantly with lower amyloid burden (β = ‐0.24, 95% CI: [‐0.42, ‐0.07], *p* = 5.65e‐03) and with greater hippocampal volume (β = 0.23, 95% CI: [0.08, 0.38], *p* = 3.73e‐03). There was also a trend‐level association between higher COPR with greater meta‐ROI cortical thickness (β = 0.20, 95% CI: [0.03, 0.37], *p* = 0.02). There was no relationship between COPR and WMH volume (Table 3).

Finally, higher composite‐COR was associated with lower global amyloid SUVR (β = ‐0.25, 95% CI: [‐0.43, ‐0.08], *p* = 5.15e‐03), and greater hippocampal volume (β = 0.26, 95% CI: [0.11, 0.41], *p* = 6.21e‐04), but not with WMH volume (**Table** **3**). Higher composite‐COR was also nominally associated with greater meta‐ROI cortical thickness (β = 0.18, 95% CI: [0.01, 0.36], *p* = 0.04).

All model‐based hemodynamic indices correlated with total MoCA scores, such that higher indices were associated with higher MoCA scores (all βs > 0.22, all ps < 0.018), but the physiological versions of the hemodynamic indices did not (all βs < 0.11, all ps > 0.10).

### 3.4 Moderating effect of cognitive performance on relationships between hemodynamic indices and neuroimaging outcomes

Formal interaction tests of MoCA by hemodynamic indices on neuroimaging measures were not significant, although analyses stratified by cognitive status showed that relationships between hemodynamic indices and neuroimaging outcomes were evident mostly in cognitively impaired individuals (Table 4).

**TABLE 4:** Results of linear regression models relating each hemodynamic index to each neuroimaging outcome separately for cognitively unimpaired and cognitively impaired participants and results of linear regressions testing for an interaction between total MoCA score and each hemodynamic index on each neuroimaging outcome. All regression models included covariates for age, sex, site, total MoCA score, and *APOE* ε4 as well as intracranial volume for analyses that use WMH or hippocampal volume as outcomes.

	Cognitively unimpaired	Cognitively impaired	Interaction with MoCA
DCA vs global amyloid SUVR	β = ‐0.07, 95% CI: [‐0.36, 0.23], *p* > 0.10, *N* = 48	β = ‐0.30, 95% CI: [‐0.59, ‐0.02], *p* = 0.04^*^, *N* = 51	DCA x MoCA on global amyloid SUVR: β = 0.16, 95% CI: [‐0.04, 0.35], *p* > 0.10, *N* = 99
COCR vs hippocampal volume	β = 0.03, 95% CI: [‐0.18, 0.25], *p* > 0.10, *N* = 61	β = 0.10, 95% CI: [‐0.12, 0.32], *p* > 0.10, N = 69	COCR x MoCA on hippocampal volume: β = ‐0.01, 95% CI: [‐0.17, 0.15], *p* > 0.10, *N* = 130
COPR vs global amyloid SUVR	β = ‐0.16, 95% CI: [‐0.43, 0.11], *p* > 0.10, *N* = 53	β = ‐0.24, 95% CI: [‐0.49, 0.02, ***p* = 0.07**, *N* = 60	COPR x MoCA on global amyloid SUVR: β = ‐0.03, 95% CI: [‐0.19, 0.12], *p* > 0.10, *N* = 113
COPR vs hippocampal volume	β = 0.04, 95% CI: [‐0.18, 0.26], *p* > 0.10, *N* = 61	β = 0.22, 95% CI: [‐0.02, 0.46], ***p* = 0.07**, *N* = 69	COPR x MoCA on hippocampal volume: β = ‐0.003, 95% CI: [‐0.12, 0.11], *p* > 0.10, *N* = 130
Composite‐COR vs global amyloid SUVR	β = ‐0.01, 95% CI: [‐0.29, 0.27], *p* > 0.10, *N* = 53	β = ‐0.26, 95% CI: [‐0.51, ‐0.01], *p* = 0.04^*^, *N* = 60	Composite‐COR x MoCA on global amyloid SUVR: β = 0.08, 95% CI: [‐0.10, 0.25], *p* > 0.10, *N* = 113
Composite‐COR vs hippocampal volume	β = 0.10, 95% CI: [‐0.12, 0.31], *p* > 0.10, *N* = 61	β = 0.16, 95% CI: [‐0.07, 0.40], *p* > 0.10, *N* = 69	Composite‐COR x MoCA on hippocampal volume: β = ‐0.004, 95% CI: [‐0.13, 0.13], p > 0.10, N = 130

p < 0.10 bold, p < 0.05 bold with asterisk.

### 3.5 Sensitivity analyses

Higher physiological COPR was associated with greater hippocampal volume (β = 0.19, 95% CI: [0.06, 0.34], *p* = 5.10e‐03), but not with global amyloid SUVR (β = ‐0.05, 95% CI: [‐0.21, 0.11], *p* > 0.10). Higher physiological COCR was associated with greater hippocampal volume (β = ‐0.14, 95% CI: [‐0.27, ‐0.002], *p* = 4.67e‐02). Finally, higher physiological DCA was not significantly associated with global amyloid SUVR (β = 0.01, 95% CI: [‐0.16, 0.19], *p* > 0.10).

## 4 DISCUSSION

In this cross‐sectional study of cognitively impaired and unimpaired older adults, we found that novel, model‐based cerebral hemodynamic markers are associated with established ADRD‐related neuroimaging measures. Our findings corroborate mounting evidence of an important role of cerebrovascular dysfunction in the pathogenesis of ADRD. Since the cerebrovascular indices examined in our study are derived from non‐invasive techniques (transcranial doppler ultrasound and near‐infrared spectroscopy) that are less expensive than MRI or PET, our findings have important implications for the potential clinical utility of such measures.

Consistent with several prior studies^30^, ^31^, ^39^ we report significant differences in these model‐based hemodynamic indices between cognitively impaired and unimpaired older adults in our study, providing further support for their potential diagnostic utility. Notably, while prior work has reported no differences in TCD‐based cerebral autoregulation between healthy adults and AD patients^12^, here we found significant differences in our TCD‐based DCA index between cognitively impaired and unimpaired participants. This may be because our stochastic approach yields statistical indices that are more sensitive to the differences in the hemodynamic characteristics between two clinical groups of interest. Furthermore, our study extends prior reports of an association between greater cerebrovascular reactivity and better cognitive performance^40^, ^41^, ^42^ to report that higher DVR, a measure of cerebral blood velocity in response to CO_2_ changes, is modestly associated with lower WMH volume. Additionally, both model‐based indices derived from near‐infrared spectroscopy, namely COPR and COCR, which capture how cortical tissue oxygenation changes in response to arterial blood pressure and end‐tidal CO_2_ fluctuations, respectively, correlated with ADRD‐related neuroimaging outcomes. Specifically, higher COPR and COCR values (and the composite COR values derived from them) were associated with lower global amyloid burden, and greater hippocampal volume. Higher COPR and composite‐COR values were also at a trend‐level associated with greater cortical thickness in an AD‐signature region.

The hippocampus may be especially vulnerable to damage resulting from cerebrovascular deficits due to the unique architecture of its microvasculature^43^, ^44^. This heightened susceptibility of the hippocampus to vascular damage may contribute to the associations we observed between vascular indices and hippocampal volume. Susceptibility to cerebrovascular deficits may extend to other medial temporal lobe structures beyond the hippocampus. In older adults, greater blood pressure variability correlates with concurrent hypoperfusion in the hippocampus, parahippocampal gyrus, entorhinal cortex and perirhinal cortex^45^. Furthermore, in cognitively impaired older adults, global hypoperfusion correlates with cortical thinning in the same temporal AD‐signature region that we found to be modestly associated with near‐infrared spectroscopy based hemodynamic indices in our study^46^.

We also observed associations between higher DCA, COPR, and composite‐COR and lower brain amyloid deposition. Animal studies demonstrate a bidirectional relationship between cerebrovascular function and amyloid processing. Mice overexpressing the amyloid precursor protein exhibit impaired cerebrovascular autoregulation and reactivity^47^, ^48^ with similar deficits observed in wild type mice exogenously treated with amyloid beta peptides^49^. Conversely, cerebrovascular dysfunction can increase amyloid accumulation through direct and indirect pathways. Direct links may involve hypoperfusion‐induced changes in amyloid precursor protein expression and cleavage^27^, ^50^, ^51^ while indirect links may involve defective amyloid clearance mechanisms or greater extravasation of circulating amyloid peptides into the brain^52^.

Similar to previous reports of more severe cerebral white matter lesions in relation to lower cerebral vasomotor reactivity^53^, ^54^ we found that lower DVR correlated with greater WMH volume though this relationship did not survive correction for multiple comparisons. In contrast, we observed no association between WMH volume and COPR, COCR, or the composite‐COR measure. These indices are derived from near‐infrared spectroscopy which probes tissue oxygenation at the lateral prefrontal cortex and may therefore be more reflective of the hemodynamic properties of capillaries and arterioles perfusing gray matter rather than the larger blood vessels adjacent to white matter. It is possible that subtle microvascular dysfunction, even in the absence of overt cerebrovascular damage that would manifest in global white matter pathology, is sufficient to impact the clearance of cerebral amyloid and contribute to neuronal damage in vulnerable regions, such as medial temporal lobe structures^55^.

## 5 LIMITATIONS

The cross‐sectional design of our study precludes evaluation of a potential longitudinal relationship between hemodynamic and neuroimaging measures. Furthermore, the directionality of the observed associations cannot be determined. While hemodynamic impairments can contribute to pathogenesis and neurodegeneration, it is possible that AD pathology and neuronal loss manifests in reduced metabolic activity and less pliant vasculature that can lead to cerebrovascular dysfunction. However, we hope to address this limitation in a follow‐up analysis, as longitudinal data collection is currently underway. Future studies should also examine relationships between hemodynamic indices and cognitive measures that are more sensitive to cognitive variability across the cognitive spectrum. Our sample consisted of mostly non‐Hispanic White older adults with low cardiovascular risk, which is not representative of the demographic and clinical profile of the wider elderly population in the United States. Future studies in samples that are more ethnically and racially diverse would be helpful for better understanding these relationships in older adults more generally. Finally, amyloid‐PET was completed by 67 out of the 88 cognitively impaired participants in our study, and of those, 47 were amyloid positive and 20 were amyloid negative and presumably have cognitive impairment due to non‐AD etiology. Consequently, these hemodynamic indices may be candidate markers of cerebrovascular health relevant to AD but also more generally to dementia.

## 6 CONCLUSION

Our study demonstrates that novel cerebral hemodynamic markers correlate with ADRD‐related neuroimaging profiles in cognitively impaired and unimpaired older adults. Importantly, our findings provide support for the use of model‐based hemodynamic markers – obtained using inexpensive, noninvasive techniques that do not require cooperative effort by the participant – to characterize hemodynamic impairments associated with MCI and dementia in older adults.

## CONFLICT OF INTEREST STATEMENT

The authors declare no conflicts of interest.

## CONSENT STATEMENT

All human subjects provided informed consent.

## Supporting information



Supporting Information

Supporting Information
