# Supplementary material for: Cerebrovascular regulation dynamics and Alzheimer's neuroimaging phenotypes
Source: Alzheimers Dement. 2026 Feb 13;22(2):e71146. doi: 10.1002/alz.71146 (PMC12902799; doi:10.1002/alz.71146)
Supplement: Supplementary file 1 — Supporting Information [file ALZ-22-e71146-s001.docx]

### Supplementary Material: Cerebrovascular regulation dynamics and Alzheimer’s neuroimaging phenotypes

**Figure S1.** Flow chart of participant inclusion and exclusion and arrival at the final study samples for each imaging modality for COCR, COPR and composite-COR analyses.

Enrolled in Study

(n=214)

COCR/COPR/composite-COR measurement obtained by time of analysis

(n=206)

MRI

(n=161)

PET

(n=136)

25 Participants excluded:

2 Incidental finding

2 Segmentation QC failure

1 Sequence not completed

20 missing MoCA

23 Participants excluded:

7 Segmentation QC failure

3 Motion

13 missing MoCA

31 Participants excluded:

11 ROI segmentation QC failure

2 Incidental finding

18 missing MoCA

SUVR

(n=113)

85 Participants excluded:

67 ROI segmentation QC failure

2 Incidental finding

16 missing MoCA

Meta-ROI

(n=76)

Hippo

(n=130)

WMH

(n=136)

**Figure S2.** Flow chart of participant inclusion and exclusion and arrival at the final study samples for each imaging modality for DVR and DCA analyses.

Enrolled in Study

(n=214)

DVR/DCA measurement obtained by time of analysis

(n=167)

MRI

(n=135)

PET

(n=123)

19 Participants excluded:

2 Incidental finding

1 Segmentation QC failure

16 missing MoCA

24 Participants excluded:

5 Segmentation QC failure

5 No MRI

2 Motion

12 missing MoCA

SUVR

(n=99)

26 Participants excluded:

9 ROI segmentation QC failure

2 Incidental finding

15 missing MoCA

69 Participants excluded:

52 ROI segmentation QC failure

2 Incidental finding

15 missing MoCA

Meta-ROI

(n=66)

Hippo

(n=109)

WMH

(n=116)

**Table S1.** Overlap between COCR, COPR, composite-COR and DVR samples with usable MRI or PET data.

| **N** | **COCR/COPR/composite-COR** | **DVR** | **DCA** | **usable MRI** | **usable PET** |
| --- | --- | --- | --- | --- | --- |
| 156 | x |  |  | x |  |
| 126 | x |  |  |  | x |
| 125 | x |  |  | x | x |
| 132 |  | x | x | x |  |
| 111 |  | x | x |  | x |
| 110 |  | x | x | x | x |
| 132 | x | x | x | x |  |
| 111 | x | x | x |  | x |
| 110 | x | x | x | x | x |

**Details regarding our quality checking protocol for regional cortical thickness data:**

Our protocol is based on ENIGMA’s Cortical Quality Control Protocol 2.0 (April 2017) for the analysis of FreeSurfer mean cortical thickness ROI data [1]. Differences between our protocol and the ENIGMA protocol are as follows:

- We evaluate the “internal” QC, which determines whether gray matter has been correctly segmented from other tissue as seen on coronal slices. The ENIGMA protocol additionally reviews “external” QC, evaluating the surface area extent of each region. We do not perform that step as our focus was specifically on mean cortical thickness. Any obviously incorrect parcellations (such as global fails) would still be excluded.
- We have altered ENIGMA’s webpage format to be able to toggle the segmentation overlay on/off over the image. This allows the rater to better evaluate the accuracy of the segmentation within the html.
- We have added pass/fail checkboxes to the webpage, ordered to match the appearance of cortical regions on the screenshots. A button allows a rater to export the QC pass/fail results to a .csv file eliminating the previous need to manually enter the pass-fail results to a separate spreadsheet, which can introduce user input error.
- We display more coronal screenshots of segmentation (32 vs. 8), which gives tighter coverage of segmentation. For our protocol, the spacing between each screenshot slice is approximately 6 mm. For ENIGMA’s protocol, the spacing between each screenshot slice ranges from 25 - 51 mm.
- Our 32 coronal screenshots run from the anterior 10% to the 87.5% posterior of the brain, whereas ENIGMA’s 8 coronal screenshots run from the anterior 10% to the 90% posterior of the brain.
- In our axial screenshots of segmentation, we cover 8 slices – running from the 32.5% superior to the 47.5% inferior of the brain (to look at the frontal pole, rostral anterior cingulate, and posterior cingulate) and 72.5% to 87.5% (to look at the temporal pole). ENIGMA’s 8 axial slice screenshots cover the superior 10% to the 90% inferior of the brain.
- Our meta-ROI includes the following bilateral regions: entorhinal cortex, fusiform gyrus, inferior temporal gyrus, middle temporal gyrus.
- Only participants who have passed the visual quality inspection bilaterally for all four meta-ROIs are included in our meta-ROI dataset.
- For each included participant, we calculate the following:
  - The meta-ROI weighted by surface area = the sum of (the average cortical thickness for each region * total surface area for that region)/total surface area of all regions.
- Once the meta-ROI cortical thickness values are calculated for each participant, we perform an additional check for any outliers. If outliers are found, the participant’s meta-ROIs are visually inspected again to ensure that the FreeSurfer segmentation looks reasonable. If the FreeSurfer segmentations are undersegmented or oversegmented, that participant is excluded from our meta-ROI dataset and the failed regions are excluded from the cortical thickness data as well.

**Details regarding our quality checking protocol for whole hippocampal volume data:**

We follow the guidelines of Boccardi et al., 2015, to ensure that hippocampal segmentations exclude the choroid plexus and fornix but include the alveus/fimbria white matter.

**Figure S3. The anterior and posterior borders of the hippocampus are defined by hyperintense lines in the sagittal slices.**

**
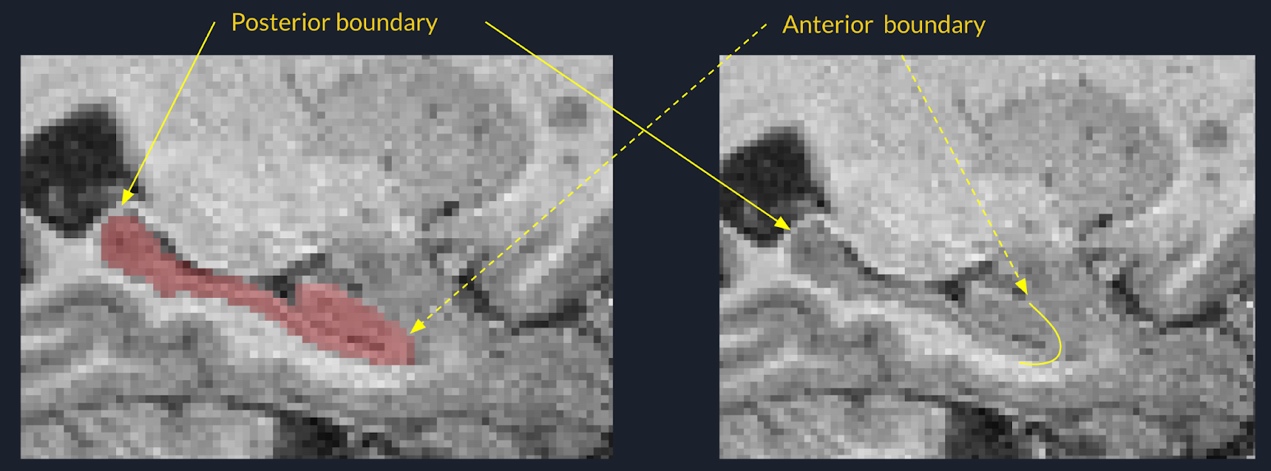
**

Using FSLview, we inspect the left and right hippocampus masks overlayed on top of the participant’s bias-corrected T1-weighted image. In the sagittal view, if there is oversegmentation (Figure S5) or undersegmentation (Figure S6) of the mask at least as large as a 2 x 2 square of voxels for 4 or more consecutive sagittal slices, the mask is a FAIL.

**Figure S4. Example of hippocampal oversegmentation.**

**
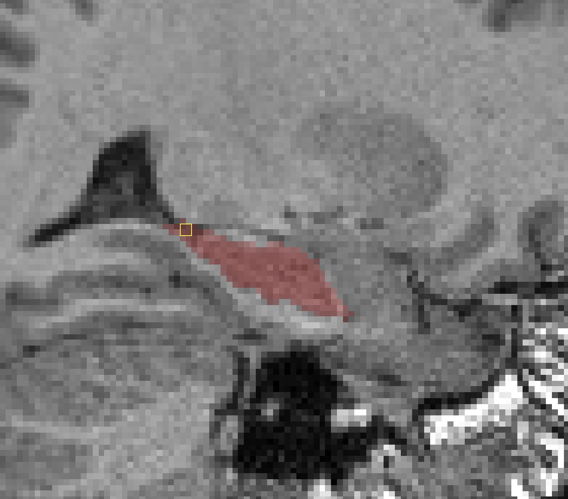
**

**Figure S5. Example of hippocampal undersegmentation.**

**
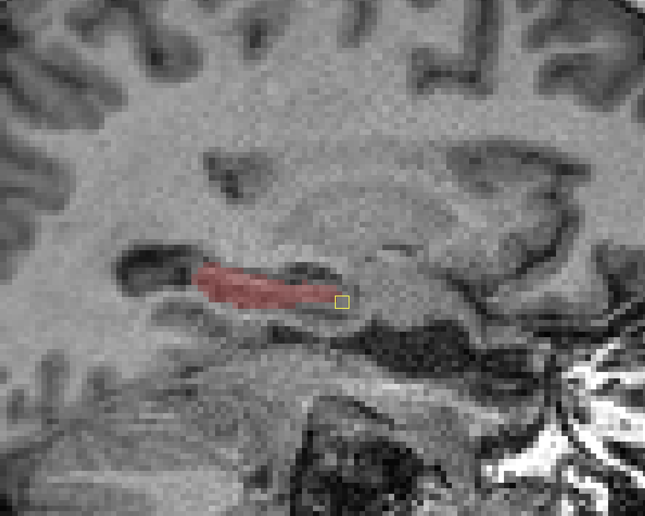
**

**Detailed methods regarding the computation of hemodynamic indices**

***Data pre-processing***

1. Signal continuity was examined visually and unusable segments of the recorded signals were excluded, while preserving the synchronicity of the various recordings. Possible outliers (due to measurement errors or artifacts) were clipped at +/- 3 SDs around the time-average value.
2. Beat-to-beat average values of the blood pressure, flow velocity and oxygenation time-series data were computed using the demarcated start and end points of each heart-beat pulse. These pulse-average values were placed at the mid-point of the respective heart-beat interval.
3. Breath-to-breath extraction of end-tidal CO_2_ was performed by identifying the maximum value of the capnography trace between the times of inhalation and exhalation. This value was placed at the mid-point of the respective breath interval.
4. The resulting asynchronous data were resampled every 0.5 s to generate evenly-sampled synchronized time-series data through cubic spline interpolation (Nyquist frequency of 1 Hz).
5. High-pass filtering was applied above 0.005 Hz in order to de-mean the data and remove very low frequencies (cycles with period >200 s) that cannot be reliably analyzed with the Laguerre expansion technique using the available 8-min input-output data-records.

***Data analysis***

The hemodynamic markers were obtained by use of a methodology that is *rigorous* (i.e. based on canonical mathematical and computational methods), *objective* (i.e. not requiring subjective judgment) and *robust* (i.e. exhibiting constrained estimation variance in the presence of heavy noise and physiological interference). To achieve this, the time-series data were analyzed in two stages:

(1) *Dynamic predictive modeling*: whereby subject-specific “kernels” of predictive dynamic models in convolutional form were estimated using the robust and general method summarized below.

This general dynamic modeling approach utilizes the two-input linear dynamic model of Eq. (1) that represents the discrete-time output signal *y(n)* in terms of the following convolutional relation involving the two discrete-time inputs *p(n)* (blood pressure time-series data) and *x(n)* (CO2 time-series data):

*y(n) = k_0_ + Σ_m_ k_p_(m) p(n-m) + Σ_m_ k_x_(m) x(n-m) + ε(n)* (1)

where the “kernels” *k_p_* and *k_x_* in the model describe fully the dynamic characteristics of each input-output pathway and *ε(n)* represents the model prediction errors. The kernel values depend on the “lag” variable *m*, which denotes the time separating each past input value from its effect upon the output at the present time. The corresponding kernel values, *k_p_(m)* and *k_x_(m)*, define the relative influence of past (or present for *m*=0) input values upon the present value of the output, according to the weighted sum indicated in the respective convolutions of Eq. (1). The summations over *m* in Eq. (1) take place over the range of the respective “kernel memory”. The kernels are also often referred to as “Impulse Response Functions” because they represent the output when the input is an impulse. Their robust estimation from noisy data (common in physiological systems) is achieved with the use of the Laguerre Expansion Technique (LET) that is briefly described below. Upon estimation of the two kernels, we can compute the model-predicted response to any given set of inputs. LET utilizes the discrete-time Laguerre expansions of the two kernels:

*k_p_(m) = Σ_j_ c^p^_j_ L^p^_j_(m)* ,  *k_x_(m) = Σ_j_ c^x^_j_ L^x^_j_(m)*  (2)

where *L^i^_j_(m)* denotes the orthonormal discrete-time Laguerre basis (*j=1,…,K_i_*) for the *i*-th input (*p* or *x*). The suitable number of Laguerre basis functions {*K_i_*} in each application is determined through a search procedure (see below). The kernel expansions of Eq. (2) transform Eq. (1) into Eq. (3) that involves *linearly* the Laguerre expansion coefficients {*c^p^_i_*} and {*c^x^_j_*}, which can be estimated from the data via least-squares regression (a simple and robust numerical procedure).

*y(n) = k_0_ + Σ_j_ c^p^_j_ v^p^_j_(n)* *+ Σ_j_ c^x^_j_ v^x^_j_(n)* *+ ε(n)* (3)

where:

*v^p^_j_(n) = Σ_m_ L^p^_j_(m) p(n-m) , v^x^_j_(n) = Σ_m_ L^x^_j_(m) x(n-m)* (4)

Following estimation of the Laguerre expansion coefficients, *{c^p^_i_}* and *{c^x^_j_},* we can construct the kernel estimates using Eq. (2). Key parameters for the application of LET are the number of employed Laguerre basis functions {*K*} and the Laguerre parameter “*α*” that defines the relaxation dynamics of the respective Laguerre basis functions for each kernel. The optimal parameter values are selected through a search procedure over a grid of values for *K* = 1,2,…,9 and *α* = 0.1, 0.125, 0.15,…,0.9 (in increments of 0.025) that seeks to minimize the *Bayesian Information Criterion (BIC)*, which takes into account the sum-of squared residuals *Q* of the model prediction for each pair of parameter values (*K*, *α*), the data-record length *N* and the number of free parameters *(K+1)* in the respective model as:

*BIC (K, α) = N ln[Q(K, α)/N] + (K+1) ln(N) (5)}{\displaystyle \mathrm {BIC} ={\ln(n)k-2\ln({\hat {L}})}.\ }*

For our two-input model, this search takes place first for the input *p* (single-input prediction of the output *y*) and, after the optimal values (*K*, *α*) have been determined for input *p*, the optimal single-input prediction is computed and subtracted from the output *y* to yield the residual output that is subsequently used in the search procedure for the input *x*. To validate the statistical significance of the estimated kernels, we used a computational “boot-strapping” method that computes “null” kernel estimates under the Null Hypothesis that there is no predictive relation between the inputs and the outputs after randomly shuffling the output time-series data. Then we use these “null” kernel estimates (1000 in this study) to generate the “null” model predictions from which the Normalized Mean-Square Error (NMSE) of each model prediction is computed (as the sum-of-squared residuals divided by the sum-of-squared demeaned output values) and used to construct the histogram of the NMSE values for the Null Hypothesis – against which, the NMSE value of the actual estimated model prediction can be tested at 5% level of significance for each subject. Application of this boot-strapping method demonstrated the statistical significance of all estimated kernels in this study.

The obtained kernel estimates naturally exhibit some variability across participants due to expected innate inter-subject variability and random influences on the input-output data from multiple sources of physiological interference (i.e. the multitude of physiological mechanisms that influence the regulation of cerebral perfusion and cortical oxygenation dynamics, which are not observable). Therefore, it stands to reason that the estimated kernel expansion coefficients { *c^p^_j_* } and { *c^x^_j_* } may not be viewed as fixed deterministic quantities across all participants in each group (e.g. patients or controls) but rather as random variables following a fixed probability distribution characteristic of each group. This distribution should be approximately Gaussian due to the Central Limit Theorem and the fact that the coefficient estimates are computed as linear combinations of long data vectors. The mean and variance of these Gaussian distributions can be estimated from the data. These thoughts also apply to the PDM gains (see below) that are linear transformations of the kernel expansion coefficients – thus the Gaussian assumption still holds. This view introduces the concept of the “*stochastic kernel*” for dynamic modeling of systems subject to stochastic unobserved influences by multiple hidden confounders (typical of physiological systems). This concept allows rigorous statistical analysis of differences in dynamic predictive models and associated physio-markers that correspond to two groups of interest (see below).

(2) *Computation of the diagnostic physio-markers*:

The computed diagnostic physio-markers represent statistical measures of how close a given participant is to the representative statistical characteristics of one group of interest vs another. These statistical measures are computed using the *log-likelihood ratio* (LLR) of the estimated dynamic characteristics of each participant in the form of the respective PDM gains (see below) with reference to the representative statistics of each group that are determined by the group data separately for *APOE* ε4 carriers and non-carriers, since it has been found that the presence of the *APOE* ε4 allele affects significantly the cerebral perfusion regulation dynamics.

The PDM gains that are used for the computation of these physio-markers are obtained as follows. The estimated subject-specific kernels are placed in a rectangular matrix that is subjected to Singular Value Decomposition (SVD) to yield the orthonormal set of Principal Dynamic Modes (PDM) for each input-output pathway of dynamic transformation as the Singular Vectors, which are ranked in importance by the respective Singular Values. Subsequently, the expansion coefficients of each kernel on the respective orthonormal PDM basis are computed via inner products and termed “*PDM gains*”. The obtained PDM gains generally vary across subjects but follow nearly-Gaussian distributions for each group under study (i.e. controls or patients). This gives rise to the aforementioned concept of a “stochastic kernel”, which is a linear combination of the respective PDMs with coefficients (the PDM gains) that are Gaussian random variables with mean and SD for the respective group estimated from all available data.

Thus, we can compute the LLR of each estimated PDM gain under the Gaussian assumption, which is a measure of the statistical proximity of the particular PDM gain to the representative statistical characteristics for the two groups A vs B. For the *i-th* PDM gain, the LLR is given by:

*LLR_i_ = - ln(s_A_) - [(g_i_ - m_A_)/s_A_)^2^]/2 + ln(s_B_) + [(g_i_ - m_B_)/s_B_)^2^]/2 (6)*

where *g_i_* is the estimated gain of the *i-th* PDM for a given input-output relation (kernel), *m_A_* and *m_B_* denote the mean values of the Gaussian distributions for groups A and B, respectively, and *s_A_* and *s_B_* denote the SD values of for groups A and B, respectively. The LLR values of the PDM gains are unitless and are clipped at +/- 4 to mitigate the effects of outliers. Positive *LLR_i_* values indicate that the *i-th* PDM gain is likely to belong to group A and, conversely, negative *LLR_i_* values indicate that it is likely to belong to group B (in a graded manner). We compute four separate physio-markers that quantify distinct aspects of the regulation of cerebral perfusion and cortical oxygenation dynamics. Each individual physio-marker is computed as the *weighted* sum of the LLRs of all PDM gains for the respective input-output pathway, with the weights being determined through regression of a dependent variable of 100 for group A or -100 for group B on the LLR values of all relevant PDM gains. The CCI physio-marker is based on *both left and right* NIRS sensor measurements by forming the weighted sum of the LLRs of all relevant PDM gains – thus representing a statistical measure of the regulation of oxygenation in both left and right regions of the prefrontal cortex in response to spontaneous changes of CO2 and blood pressure at resting conditions. All weights used for the computation of each physio-marker remain fixed for all participants. Therefore, these physio-markers represent *statistical measures* of how close a given participant is to the representative statistics of one group of interest vs another.

**References**

[1] Imaging Protocols. ENIGMA. Accessed December 3, 2024. <https://enigma.ini.usc.edu/protocols/imaging-protocols/>

### [2] Boccardi, Marina, et al. “Training labels for hippocampal segmentation based on the EADC‐ADNI Harmonized Hippocampal protocol.” *Alzheimer’s and Dementia*, vol. 11, no. 2, 20 Jan. 2015, pp. 175–183, https://doi.org/10.1016/j.jalz.2014.12.002.
